# Cats and dogs: Best friends or deadly enemies? What the owners of cats and dogs living in the same household think about their relationship with people and other pets

**DOI:** 10.1371/journal.pone.0237822

**Published:** 2020-08-26

**Authors:** Laura Menchetti, Silvia Calipari, Chiara Mariti, Angelo Gazzano, Silvana Diverio

**Affiliations:** 1 Laboratorio di Etologia e Benessere Animale (LEBA), Dipartimento di Medicina Veterinaria, Università degli Studi di Perugia, Perugia, Italy; 2 Dipartimento di Scienze Veterinarie, Università di Pisa, Pisa, Italy; Faculty of Animal Sciences and Food Engineering, University of São Paulo, BRAZIL

## Abstract

Although popular culture describes them as mortal enemies, more and more often, dogs and cats live under the same roof. Does this make them best friends? Can sharing the same social and physical environment make them similar? This study compares the approaches of dogs and cats living in the same household have towards humans and other pets as perceived by the owner. Questionnaires collected from 1270 people owning both dog(s) and cat(s) were analysed. Most dogs and cats living together are playful with familiar humans (76.2%) but dogs have a more sociable approach towards strangers and conspecifics than cats (P<0.001). Moreover, the percentage of dogs that have a playful relationship with the owner (84.0%) was higher than cats (49.2%; P<0.001). Dogs and cats living together eat in different places and show different mutual interactions: more dogs lick the cat (42.8%) and more cats ignore the dog (41.8%) than vice versa (P<0.001). However, most dogs and cats sleep at least occasionally (68.5%) and play together (62.4%; P<0.001). Although some body postures, such as the tail’s position, are interpreted differently by the two species, the greater proportions of dogs and cats show a relaxed response to several kinds of approaches of their roommate. Our questionnaire confirms the common beliefs about the sociability of the dog and the privacy of the cat, but this does not result in continuous internal struggles. Most cohabitations are peaceful. Moreover, it is true that they speak different languages, but they seem to understand each other well and interpret each other's approaches in the right way. Thus, aspiring owners should not blindly believe popular assumptions, but both knowledge and respect for species-specific pet behaviours are essential to establish a balance in the household.

## Introduction

In the last 9.000 years, cats and dogs have been living in settlements with humans and assuming crucial roles within the communities. Dogs, the earliest to be domesticated, proved useful as guards, herding and as hunters [1]. To date, the dog is also appreciated for its ability to detect people, explosives or drugs, and to assist humans with disabilities [2–6]. The cat was domesticated later and it has played an important role in controlling rodents [1] though contemporary society especially appreciates its ease of management [7]. Both the cat and the dog are today of priceless value also as companions. They provide amusement and pleasure, they encourage social interactions, alleviate loneliness and comfort at stressful times, separations or mourning, as well as bring benefits in various pathological conditions [4, 8, 9].

For all these and other reasons, to date about one in four people owns at least a dog or a cat (data referring to America and Europe [10, 11]) and many had both species [12, 13]. A survey in the United Kingdom indicated that 7% of respondents had both species [14] while, among the participants of our previous survey in Italy, this percentage rose to 43% [12].

Owners consider their dog or cat to be family members [12, 15] but perceive differences in the personality of the two species: dogs prove more sociable and protective whilst cats more neurotic [16, 17]. Physiological and ethological features, as well as divergent evolutionary trajectories, may have modulated dogs and cats’ social behaviours [17–19]. Moreover, the sensitivity, the prejudices, and the attachment level of the observer could affect the qualitative judgment on pets’ behaviour [13, 17]. Dog adopters have higher expectations for the behaviour and the relationship with their pet compared to cat adopters [20]. Moreover, the tendency to consider their pet as “family member” is weaker in cat owners than dog owners [15].

Actually, both dogs and cats show interspecies communicative skills [21–24] and manifest attachment behaviour towards their owner [25, 26]. In addition to the olfactory sense, both dogs and cats use body postures, facial expressions and vocalizations to communicate [27–29]. Nevertheless, dog and cat’s language may be different and this could influence their mutual relationship, as well as those with humans and with other animals. Studies on interrelationships of dogs and cats report that dogs use more referential gazes towards humans compared to cats, while the latter display more aggressive or antagonizing behaviours towards dogs than vice versa [7, 13, 23]. The knowledge of the comparative aspects of expressive language could improve human-pet interactions, animal welfare, and management especially in the multiple-pets homes [13, 17]. It is also interesting to understand if and how the owner perceives these differences. As mentioned above, the perception of such differences could be influenced by popular breed stereotypes and owner’s expectations and, in turn, could also imply a different approach of the owner towards the two species. For example, the owner can expect that cats behave similarly to dogs or expect that the cats have the same body language that dogs have. It would lead to negative interpretations of unexpected (but normal) cat behaviours.

Little has been studied on the coexistence of dogs and cats under the same roof. This social phenomenon is growing [7, 13, 17] and offers interesting topics of study. For instance, this peculiar situation allows to evaluate the approach of a pet with both known and unknown animals and if pets that share the same environmental and social context have similarities in setting up their relationships. Moreover, dogs and cats are judged by the same owner, therefore, reducing the possible influences related to the demographic characteristics and the sensitivity of the owner.

Then, we investigated the intra and interspecies relationships of dogs and cats by administering a questionnaire to owners of both species. Specifically, we analysed how the owner perceives the approach of dogs and cats towards people and conspecifics as well as dog-cat interactions. We also investigated the differences in attitudes according to the familiarity that pets have with people or other animals. We hypothesized that, although we could not directly assess the behaviour of cats and dog living in the same household, we might still provide reliable and valid information regarding this behaviour by collecting the owners’ perceptions and opinions about it. Thus, this has to be regarded as a study based on owners’ reports and not on dog and cat behaviour.

## Materials and methods

### Participants

The questionnaire was voluntarily and anonymously completed by 1270 people owning at least one dog and one cat. This sample can be considered representative of a larger population. Data were collected over three-month period (from May to July 2014) from 1270 residents in Italy, who voluntarily and anonymously completed the questionnaire. We could not calculate the relative response rate because the questionnaire was distributed through email and social networks.

### Ethical statement

All participants had to provide their written consent and they were briefly debriefed before answering the questionnaire. All of them anonymously and voluntarily agreed to participate in the study. In our study, the questionnaire did not collect any privacy-sensitive data, identifying information or health information of the participants, items were invented by the authors and did not aim at assessing respondents’ personality. Our focus was on the animals, but none of them has been used for the research. No one animal has been treated in any way or submitted to any experimental protocol. Thus, ethical approval was not required.

### Materials and procedure

The study is part of a broader project, named “RandAgiamo^®^”, which aims to increase the adoptability of adult shelter dogs in the Umbria region of Italy (for details see [30, 31]).

The survey was carried out by using an online questionnaire (Google Form^®^). The link was spread via email (to authors' acquaintances) via various cat and dog breed-specific groups, public and through social media e.g. Facebook to generate a snowball sample. The questionnaire was preceded by an introduction explaining the purpose of the study, the characteristics required to participate in the survey (being 18 years or older and having owned at least a dog and a cat at the same time), and the instructions on how to fill it out. In particular, it was specified that if the participant had more than one dog/cat, the answers had to be referred to the first pet of each species acquired still living at home. The questionnaire also included a sentence with which respondents were giving their consent to the collection of data, which were collected anonymously and treated according to the Italian law on privacy.

In the present work, we analysed only a subset of this long-form questionnaire, which explored several aspects of the cohabitation between dogs and cats (for details see [17]).

The part of the questionnaire analysed in this paper concerns the dog-cat relationship and was divided into five sections, as follows:

Section A included 5 multiple choice questions on participant demographic data (age, gender, region of residence, previous pet-ownership, specific expertise on pets, number of pets owned).

Section B consisted of multiple choice questions concerning the physical characteristics (age, sex, de-sexing status, type, health status) and age of acquisition of the dog. To facilitate the comparison between dogs and cats, age and age at acquisition were categorized as shown in S1 Table.

In Section C the participants had to describe their dog’s behaviour during previous meetings with cats and vice versa (approach when the dog saw a cat, whether the dog was attacked and/or injured by a cat,). To determine the relationship with other known and unknown conspecifics, possible behavioural approaches were given in the questionnaire as follows: (a) Playful / Sociable, (b) Uninterested, (c) Aggressive, (d) Scary / Suspicious, (e) Other.

Section D-E: contained the same questions as Sections B and C, respectively, but about the cat.

Section F contained questions related to the relationship between cohabiting dogs and cats during mealtime, play-time, on returning home from the walk, while sleeping or being pampered. We distinguished the questions into those regarding the dog’s approaches towards the cat (dog-cat relationship) and the cat’s approaches towards the dog (cat-dog relationship). For interspecies interrelationship, participants could choose one or more of the following behavioural approaches: (a) licks, (b) plays, (c) ignores, (d) moves away, (e) runs away, (f) growls/hisses, (g), attacks. Finally, owners were asked to describe their dog’s behaviour in response to different postures of their cat and vice versa. For the dog’s reaction to different postures of a non-cohabiting cat, participants could choose: (a) moves away, (b) turns his head, (c) wags tail, (d) stays quiet, (e) growls (f) attacks. For the cat’s reaction to different postures of a non-cohabiting dog, the possible answers were: (a) moves away, (b) gets close amicably, (c) stays quiet, (d) hisses (e) attacks.

### Statistical analysis

The data obtained from the questionnaires were entered in an Excel sheet and transferred onto the statistical program SPSS Statistics version 23 (IBM, SPSS Inc., Chicago, IL, USA) for analysis. We used Microsoft Excel 2007 to build polar graphics. Descriptive statistics were used to present the demographic characteristics of participants and animals. Distributions within categorical variables were analysed using Chi-Square Goodness of Fit Tests. The goal was to assess whether, for each question, the answers occurred with equal frequencies and the null hypothesis assumed that there is no significant difference between the observed and the expected value (all categories equal). We used Pearson Chi square to compare the answers for dogs and cats. The null hypothesis was that the frequency of a response is not significantly different between dogs and cats. Moreover, when the owner had the same set of answers for the dog and cat, we considered the data as paired and assessed the agreement in owners’ perceptions about their cohabiting dog and cat using the McNemar or Bowker tests. The null hypothesis was that there is no difference in the response of the same owner between dog and cat (i.e. consistency in the answers that the same owner gave for its cat and dog). Only questionnaires with valid responses on both dog and cat questions were included in the comparison analysis.

## Results

### Demographic characteristics of participants, dogs and cats

Tables 1 and 2 present demographic characteristics of participants, dogs and cats (descriptive statistics). For a detailed description, please refer to Menchetti et al. [17].

**Table 1:** Demographic characteristics, age at the time of the acquisition, and living and sleeping habits of dogs (N = 1270) and cats (N = 1270) living in the same household.

Parameter		Dog	Cat
**Age**	**0–6 months**	23 (1.8%)	52 (4.1%)
	**>6 months to 2 years**	264 (20.8%)	315 (24.9%)
	**>2 to 8 years**	621 (49.0%)	561 (44.3%)
	**>8 years**	359 (28.3%)	338 (26.7%)
**Sex**	**Male**	560 (44.3%)	622 (49.2%)
	**Female**	705 (55.7%)	643 (50.8%)
**Reproductive status**	**Entire**	545 (43.3%)	138 (10.9%)
	**Neutered**	715 (56.7%)	1130 (89.1%)
**Breed**	**Mixed**^a^	664 (54.6%)	1060 (91.8%)
	**Purebred**	553 (45.4%)	95 (8.2%)
**Age with mother**	**<1 week**	31 (2.4%)	81 (6.4%)
	**Until 1 month**	219 (17.2%)	344 (27.3%)
	**Until 3 months**	572 (45.0%)	428 (34.0%)
	**>3 months**	10 (0.8%)	7 (0.6%)
	**Unknown**	438 (34.5%)	398 (31.6%)
**Age at acquisition**	**1–3 months**	736 (58.7%)	900 (72.7%)
	**4 months to 1 year**	309 (24.7%)	247 (20.0%)
	**1–8 years**	173 (13.8%)	85 (6.9%)
	**>8 years**	35 (2.8%)	6 (0.5%)
**Where the pet lives**	**Outdoors**	496 (39.4%)	69 (5.5%)
	**Indoors**	661 (52.5%)	588 (46.7%)
	**Outdoors and indoors**	103 (8.2%)	603 (47.9%)
**Where the pet sleeps**	**Free outdoors**	77 (6.1%)	95 (7.5%)
	**Enclosed space**	72 (5.7%)	53 (4.2%)
	**Home area**	168 (13.3%)	163 (12.9%)
	**Free in the home**	448 (35.4%)	599 (47.5%)
	**Bedroom**	253 (20.0%)	77 (6.1%)
	**On the bed**	232 (18.3%)	201 (15.9%)
	**Other**	16 (1.3%)	74 (5.9%)

^a^ including European cats.

Values are numbers and percentages of valid responses (in brackets).

**Table 2:** Demographic characteristics of the participants (N = 1270).

Characteristics		N°	Valid percent
**Sex of respondent**	**Men**	111	8.8%
	**Women**	1157	91.2%
**Age of respondent (class)**	**18–25 years**	217	17.1%
	**26–40 years**	559	44.1%
	**41–55 years**	400	31.5%
	**56–70 years**	93	7.3%
**Region**	**North**	554	44.6%
	**Centre**	477	38.4%
	**South**	212	17.1%
**Expert**	**No expertise**	249	19.9%
	**Dogs expertise**	120	9.6%
	**Cats expertise**	62	4.9%
	**Dogs/Cats expertise**	822	65.6%
**N° dogs**	**1 dog**	686	56.4%
	**2–5 dogs**	493	40.5%
	**>5 dogs**	38	3.1%
**N° cats**	**1 cat**	446	36.9%
	**2–5 cats**	592	48.9%
	**>5 cats**	172	14.2%

Values are numbers and percentages of valid responses (in brackets).

### First encounters between dogs and cats living in the same household

For many pets, the first meeting with the other species took place before they turned six months old (55.8% and 58.0% in dogs and cats, respectively), followed by more than two years (24.5% and 22.4% in dogs and cats, respectively), and between six months and two years (19.6% and 19.5% in dogs and cats, respectively; N = 1263 dogs and 1244 cats; Chi-Square Test of Independence, χ^2^ = 1.7, P > 0.05).

According to owners’ reports, 274/1267 dogs (21.6%) attacked a cat while 304/1260 dogs (24.1%) were attacked by a cat.

### Relationship with humans of dogs and cats living in the same household

**Fig 1:**
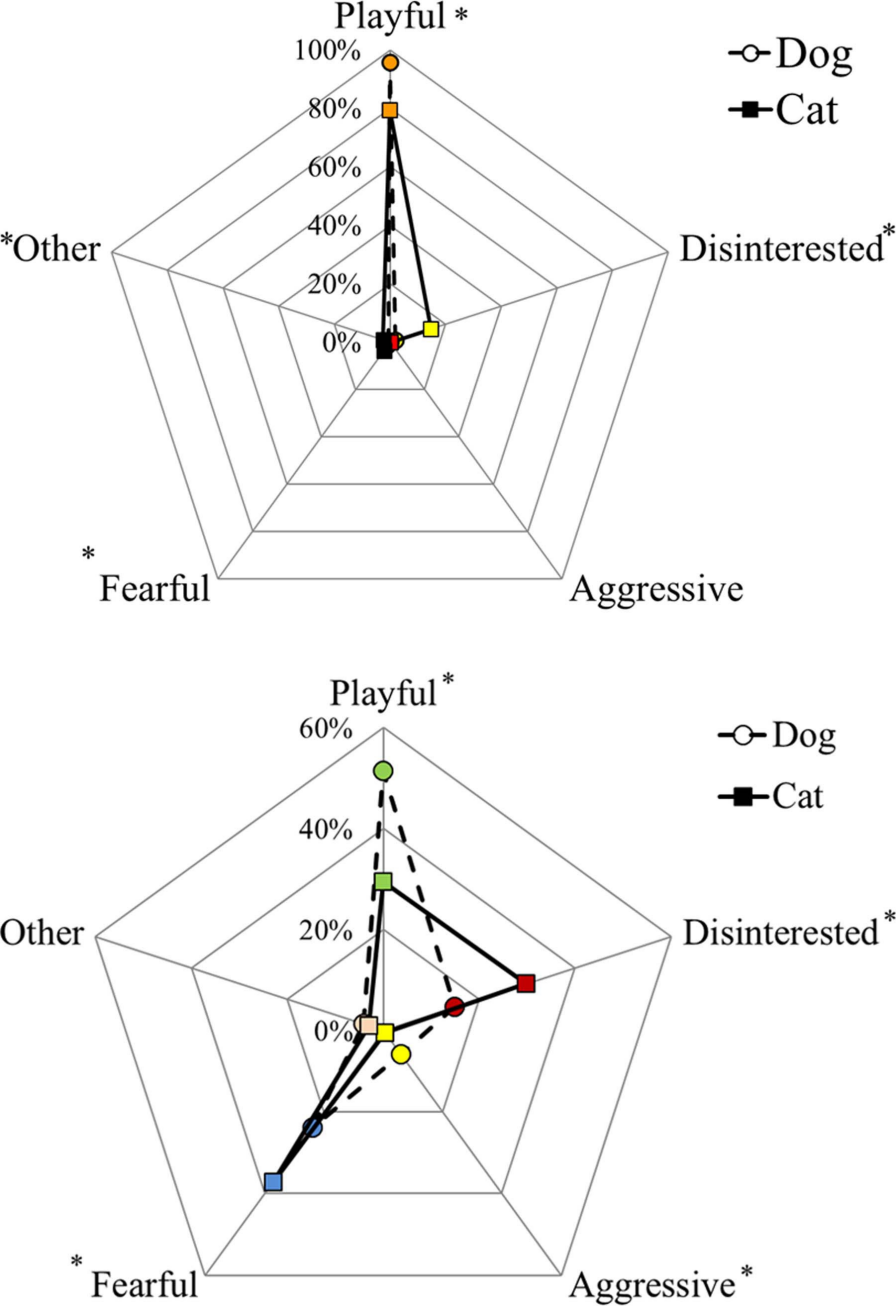
Relationship with known (a) and unknown (b) people of dogs and cats living in the same household. The asterisks indicate significant differences between dog and cat for each approach (P < 0.05).

More dogs were perceived by their owners as sociable with familiar people than cats (95.6% and 79.2% for dogs and cats, respectively), while more cats were reported as disinterested (2.0% and 14.6% for dogs and cats, respectively) or fearful compared to dogs (1.5% and 3.6% for dogs and cats, respectively; N = 1260 dogs and 1258 cats; Chi-Square Test of Independence, χ^2^ = 163.5, P < 0.001; Fig 1A). We found agreement in the perception of owner about his/her cohabiting dog and cat in 76.9% of dyads: 76.2% of dogs and cats living together were both playful with known humans, 0.5% were disinterested, and 0.2% (2 dyads) were fearful (N = 1251; Bowker test, χ^2^ = 160.4, P < 0.001).

More dogs were sociable (51.4% and 29.4% for dogs and cats, respectively) or aggressive (5.9% and 0.7% for dogs and cats, respectively) towards unknown people while cats were most fearful (23.7% and 37.1% for dogs and cats, respectively) and disinterested (14.8% and 29.8% for dogs and cats, respectively; N = 1243 dogs and 1253 cats; Chi-Square Test of Independence, χ^2^ = 227.2, P < 0.001; Fig 1B). Only 33.2% of dogs and cats living in the same household showed the same approach towards strangers (N = 1230; Bowker test, χ^2^ = 225.4, P < 0.001): 17.6% were playful, 5.9% disinterested, and 9.5% were both fearful. There were playful dogs that lived with disinterested (25.2%) or fearful (37.4%) cats, but also 71 fearful dogs living with playful cats (24.6%).

### Relationship with conspecifics of dogs and cats

**Fig 2:**
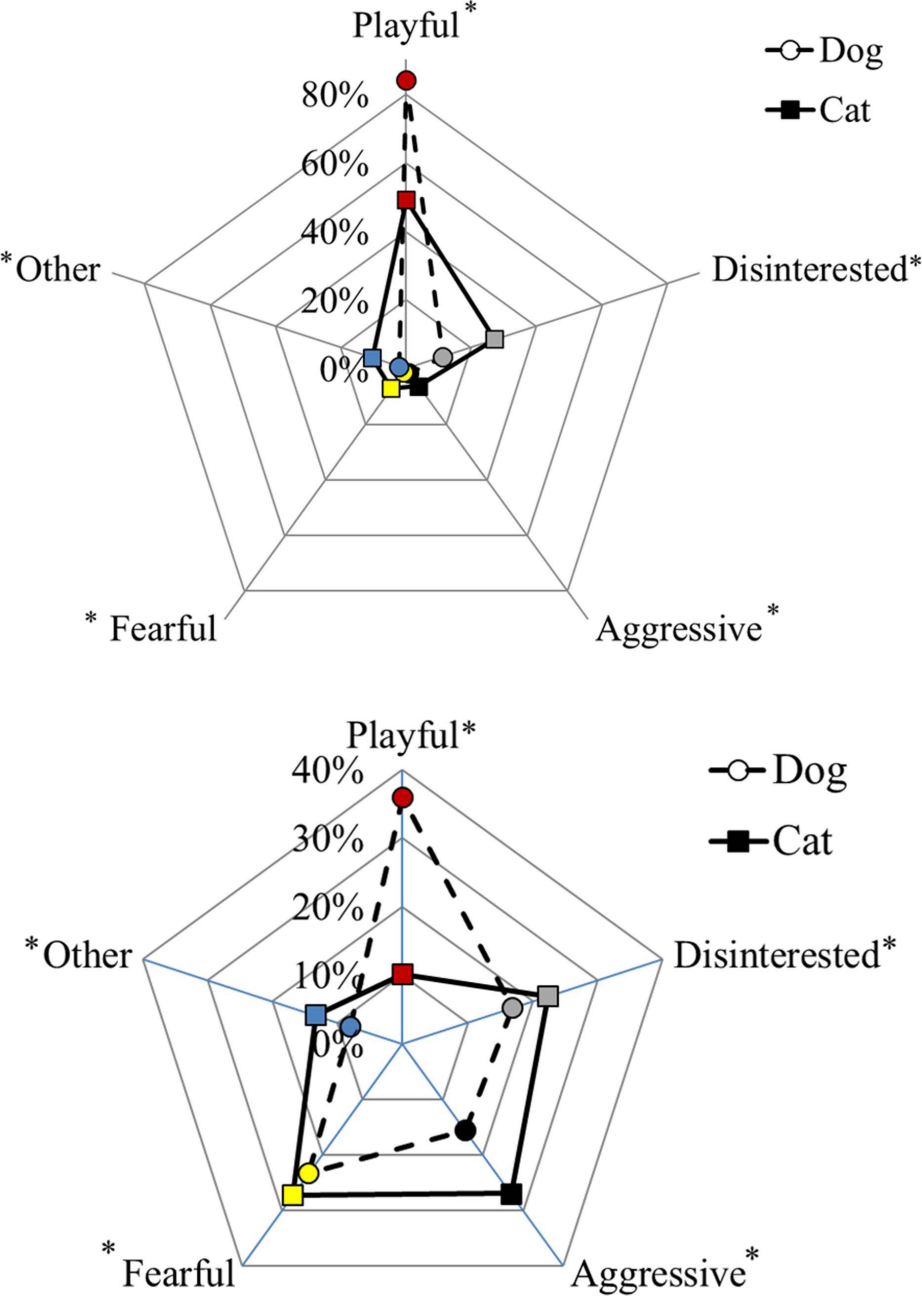
Relationship with known (a) and unknown (b) conspecifics of dogs and cats living in the same household. The asterisks indicate significant differences between dog and cat for each approach (P < 0.05).

As shown by the different shapes that the distributions take within the radar charts, dogs and cats have a different relationship with the conspecifics (Fig 2). In particular, the percentage of dogs having a playful relationship with known (84.0%; N = 1256 dogs and 1243 cats; Chi-Square Test of Independence, χ^2^ = 355.1, P < 0.001; Fig 2A) and unknown (35.9%; N = 1244 dogs and 1229 cats; Chi-Square Test of Independence, χ^2^ = 244.8, P < 0.001; Fig 2B) conspecifics was higher than cats (49.2% and 10.1% with known and unknown, respectively). More cats had a disinterested (27.3% and 22.3% with known and unknown, respectively), aggressive (6.2% and 27.0% with known and unknown, respectively), or a fearful (7.2% and 27.3% with known and unknown, respectively) approach with known and unknown conspecifics than dogs (disinterested: 11.3% and 16.9%; aggressive: 1.5% and 15.7%; fearful: 1.2% and 23.4% with known and unknown, respectively; P < 0.05; Fig 2A and 2B).

As with regards to the agreement in owner’ perception about his/her cohabiting pets, 42.7% of participants described the relationship with known conspecifics of their dog and cat as playful. However, statistics did not confirm consistency between dogs and cats of the same owner (N = 1231; Bowker test, χ^2^ = 324.8, P < 0.001). For example, dogs that had a playful relationship with other dogs could live with cats showing a disinterested (21.8%), aggressive (5.0%) or fearful (6.7%) approach towards other cats.

Relationships with unknown conspecifics are also diversified between dogs and cats living in the same household (N = 1212; Bowker test, χ^2^ = 248.0, P < 0.001) since the agreement was only found in 21.2% of participants (4.5% playful, 4.9% disinterested, 5.4% aggressive, and 6.4% fearful). 12.3% of playful dogs lived in the household with playful cats towards unknown conspecifics, while 22.4% of playful dogs lived with disinterested ones, 24.9% with aggressive and 28.5% with fearful cats towards unknown cats. We also found fearful dogs living with aggressive cats towards unknown conspecifics (26.2%).

### Response of the dog at the sight of a cat and vice versa

Participants were asked to indicate their dog's reaction to the sight of a cat and their cat's reaction to the sight of a dog. When many of the dogs saw a cat, they attacked (37.2%), barked (23.8%) or wagged their tails (20.5%; P < 0.001; Table 3). Only one dog ignored them (0.1%). On the contrary, many cats at the sight of a dog, escaped (31.3%), ignored (24.5%), hissed (21.4%) or approached him amicably (13.5; P < 0.001; Table 3). Small percentages of cats observed or attacked other dogs.

**Table 3:** Reaction of the dog at the sight of a cat and vice versa.

Item		Number and percentage	χ^2^	P value
**When the dog sees a cat, the dog…**^a^				
	**Ignores it**	1 (0.1%)	1320.5	<0.001
	**Wags his tail**	*260 (20*.*5%)*		
	**Barks**	*302 (23*.*8%)*		
	**Growls**	*162 (12*.*8%)*		
	**Chases it**	11 (0.9%)		
	**Attacks**	*473 (37*.*2%)*		
	**Runs away**	46 (3.6%)		
	**Other**	0 (0.0%)		
**When the cat sees a dog, the cat…**^b^				
	**Ignores it**	*305 (24*.*5%)*	989.0	<0.001
	**Approaches him amicably**	*168 (13*.*5%)*		
	**Hisses**	*266 (21*.*4%)*		
	**Attacks**	21 (1.7%)		
	**Observes it**	27 (2.2%)		
	**Escapes**	*390 (31*.*3%)*		
	**Other**	68 (5.4%)		

The prevailing category on the total number of respondents is indicated in Italics (Chi-Square Goodness of Fit Tests; null hypothesis: all categories equal).

^a^ Total number of respondents = 1270.

^b^ Total number of respondents = 1245.

### Relationship between the dog and the cat: Feeding and sleeping habits

Most owners (42.2%) declared that their dog ate in a bowl placed on the ground while their cat in a bowl positioned on the top of something (P < 0.001; Table 4); 27.2% of dogs and cats living in the same household ate in bowls positioned far from each other while 20.8% in bowls close to one another. In most of the dyads, the animal that finished the first the meal moved away or waited close to the other pet that was still eating (P < 0.001; Table 4).

**Table 4:** Relationship between the dog and the cat: Feeding and sleeping habits.

Item		Number and percentage	χ^2^	P value
**The dog and the cat eat…**^a^				
	**In the same bowl**	49 (3.9%)	627.2	<0.001
	**In bowls close to one another**	*260 (20*.*8%)*		
	**In bowls positioned far from each other**	*341 (27*.*2%)*		
	**The dog eats in a bowl placed on the ground while the cat in a bowl positioned at the top**	*528 (42*.*2%)*		
	**Other**	74 (5.9%)		
**The animal that ends the meal first…**^b^				
	**Moves away**	*697 (57*.*7%)*	1764.8	<0.001
	**Waits close to the animal that is still eating**	*313 (25*.*9%)*		
	**Removes the other animal and eats his meal**	88 (7.3%)		
	**Dog and cat eat together from the bowl where it remained the food**	10 (0.8%)		
	**Dog and cat eat separate**	75 (6.2%)		
	**Other**	24 (2.0%)		
**Dog and cat sleep nearby…**^c^				
	**Never**	*383 (31*.*5%)*	16.7	< 0.01
	**Occasionally**	*703 (57*.*8%)*		
	**Always**	130 (10.7%)		

The prevailing category on the total number of respondents is indicated in Italics (Chi-Square Goodness of Fit Tests; null hypothesis: all categories equal).

^a^ Total number of respondents = 1252.

^b^ Total number of respondents = 1207.

^c^ Total number of respondents = 1249.

Most cats and dogs that live under the same roof slept together occasionally (57.8%) or always 10.7% (P < 0.01; Table 4).

### Relationship between the dog and the cat living in the same household: Playing habits, interactions and mutual approaches when the dog comes back home from the walk

Most of the dogs and cats living in the same household played together (62.4%; N = 1232; Chi-Square Goodness of Fit test, χ^2^ = 702.2, P < 0.001): 58.1% chased each other, 40.9% fought, 43.9% of cats played with the dog’s tail, 63.8% made ambushes (more than one answer was possible).

**Fig 3:**
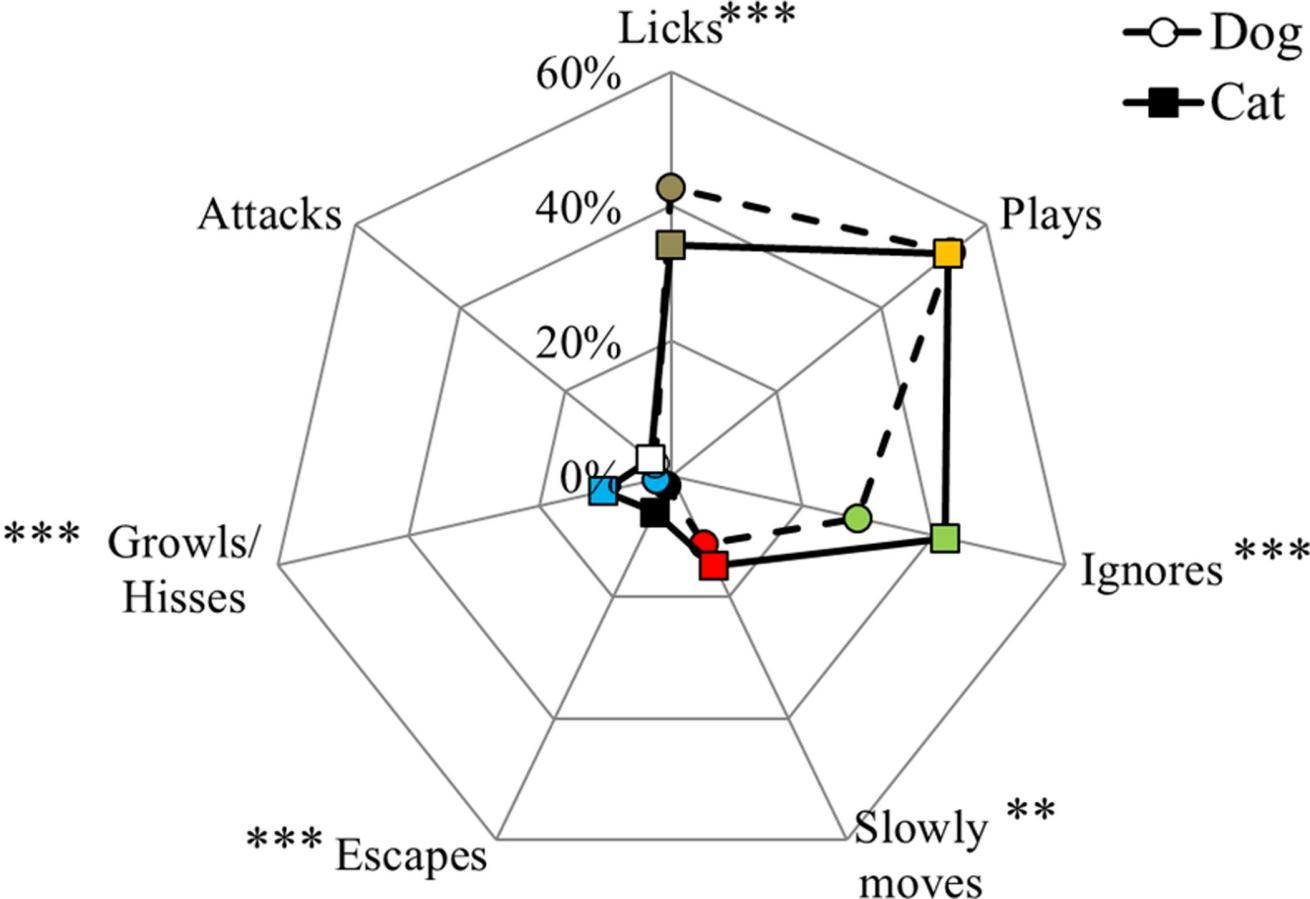
How the dog interacts with the cat (dashed line) and vice versa (solid line). The asterisks indicate significant differences between dog and cat for each approach (*P<0.05, **P<0.01, ***P<0.001).

Participants had to indicate how the dog interacted with the cat and vice versa (Fig 3). As the participants could choose more than one answer, each answer was compared between dog and cat with a Chi-Square Test of Independence (N = 1237 dogs and 1231 cats). Many more dogs licked the cat than the opposite (42.8% and 34.3% for dogs and cats, respectively; χ^2^ = 19.1, P <0 .001). On the contrary, more cats ignored (28.4% and 41.8% for dogs and cats, respectively; χ^2^ = 49.1, P < 0.001), ran away (1.5% and 5.9% for dogs and cats, respectively; χ^2^ = 6.8, P < 0.001) and hissed at the dog (2.3% of dogs growled and 10.2 of cats hissed; χ^2^ = 66.1, P<0.001) than vice versa. There was no difference between dogs and cats in the play approach (53.3% and 52.7% for dogs and cats, respectively; χ^2^ = 0.1, P = 0.783) and in the aggressive behaviours (3.1% and 3.9% for dogs and cats, respectively; χ^2^ = 1.3, P = 0.262).

Concerning the comparison of pets that live together (McNemar test; N = 1219), 24.4% of dogs and cats living in the same household licked each other (P < 0.001). Only 17.9% of dogs and cats living in the same household ignored each other (P < 0.001), 2.7% of both dogs and cats slowly moved when meeting their roommate (P < 0.05). Only 9 owners (0.7%) claimed that both their dog and cat had reciprocal aggressive vocalizations (the dog growled at the cat and the cat hissed at the dog; P < 0.001) and 6 owners (0.5%) reported that their pets attacked (P > 0.1). This last subgroup was further investigated to evaluate whether the mutual aggressive approaches reported by these 6 owners were associated with some demographic variables of the owners or of the pets, but no significant associations were found (S1 Table).

**Fig 4:**
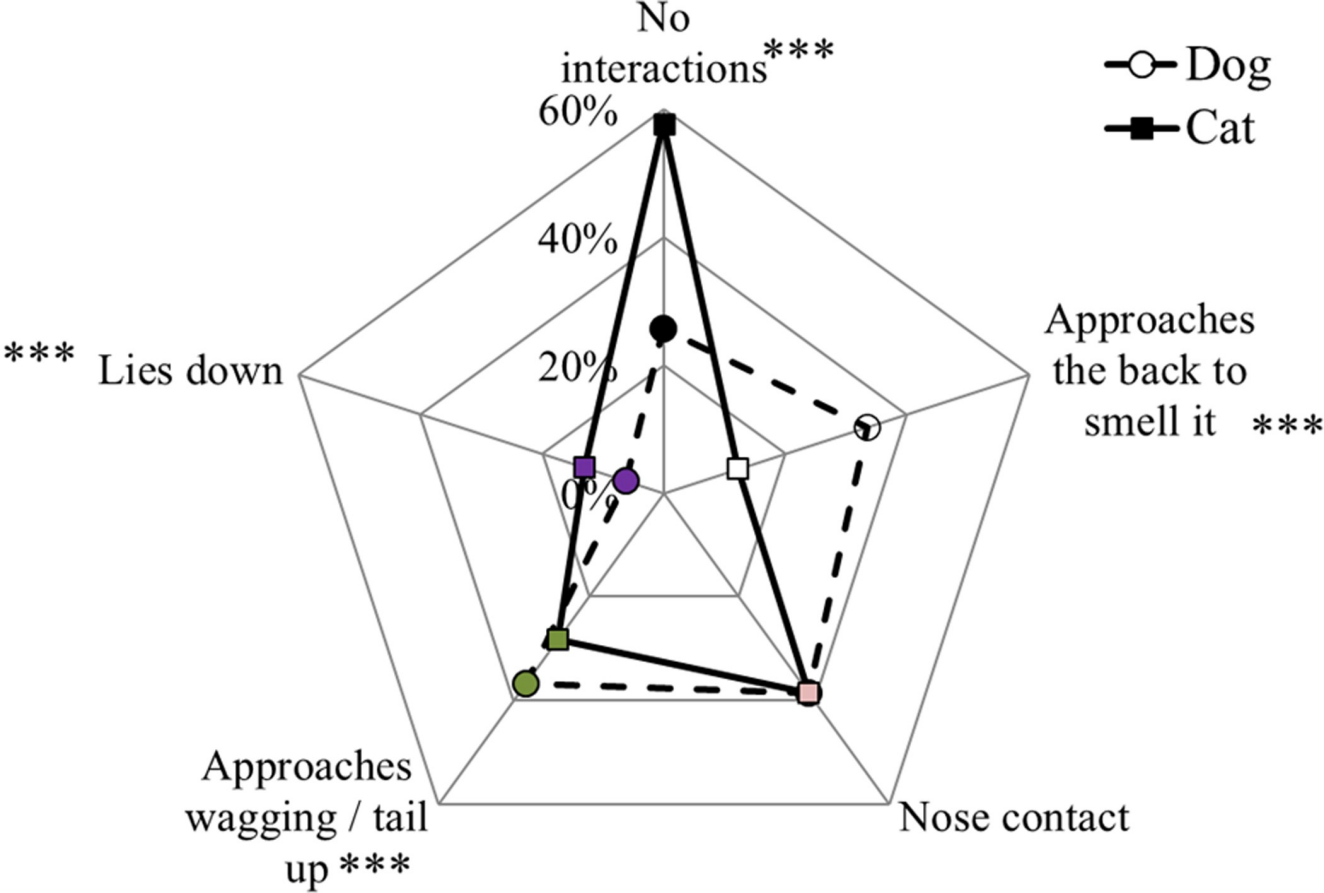
Dog (dashed line) and cat (solid line) approach to the return of the dog from the walk. The asterisks indicate significant differences between dog and cat for each approach (***P<0.001).

Owners were asked to indicate the mutual approach of their pets when the dog returned home from the walk (more than one answer was possible; Fig 4). Comparing the species (Chi-Square Test of Independence; N = 1210), there was a lower percentage of dogs that did not interact with the cat after the walk than vice versa (25.7% and 57.7% for dogs and cats, respectively; χ^2^ = 258.7, P < 0.001) but more dogs approached the back of the cat than the opposite (33.4% and 12.2% for dogs and cats, respectively; χ^2^ = 157.6, P < 0.001) and more dogs approached the cats wagging their tails than cats approaching the dog with the tail up (36.7% of dogs wagged their tails and 28.2% of cats approached with the tail up; χ^2^ = 20.2, P < 0.001). There were more cats lying down than dogs (6.2% and 13.1% for dogs and cats, respectively; χ^2^ = 34.0, P < 0.001). There was no prevalence of species for a nose contact (38.6% and 38.6% for dogs and cats, respectively; χ^2^ = 0.0, P>0.1).

### Relationship between the dog and the cat: Response of the dog to the cat's approach and vice versa

Owners were asked to describe the behaviour of their dog in response to different approaches of the cohabiting cat and vice versa. Table 5 summarizes the results while numbers, percentages and statistics are reported in the supplemental material (S2 and S3 Tables). For each question, just one answer was possible. The greater proportions of dogs and cats showed a quiet attitude (“Wags tail”, “Gets close amicably” or “Stays quiet”) in response to most hypothesized approaches. We can only notice a different distribution of percentages in response to “Approaches the dog/cat bowl”, when 25.7% of the dogs growled while 20.9% of cats moved away.

**Table 5:** Response of the dog to the cat’s approach and vice versa: Summary of the results.

CAT / DOG APPROACH	DOG RESPONSE	CAT RESPONSE
**Bends on the front limbs**	*✓ or O*	*✓ or O*
**Approaches for a nose-to-nose greeting**	*✓*	*-*^1^
**Turns his head to one side**	*O*	*O*
**Lies down beside**	*✓*	*✓*
**Wags tail**	*O*	*✓ or O*
**Approaches with tail up**	*✓*	*O or X*
**Come in the bed of dog/cat (empty)**	*O or X*	*O*
**Come in the bed of dog/cat while he sleeps**	*O or X*	*O or X*
**Approaches the dog/cat bowl**	*O or X*	*O or X*
**Approaching while the owner is cuddling the other dog/cat**	*✓*, *O or X*	*O or X*
**The cat/dog is pampered by the owner**	*✓*, *O or X*	*✓ or O*

The reaction was classified according to the prevailing responses of the owners:

*✓* = Amicable (Wags tail, Get close amicably).

*O* = Indifferent (Moves away, Turns his head, Stays quiet).

*X* = Aggressive (Growls, Attacks).

^1^ approach not considered for the dog.

Numbers, percentages, and statistics are detailed in the supplemental material (S1 and S2 Tables).

The dog wagged his tail mainly when the cat bent on the front limbs, approached him for a nose-to-nose greeting or with the tail up, lay down beside him, when the cat approached while the dog was being pampered by the owner or the owner was pampering the cat. Conversely, the dog growled or attacked mainly when the cat got in his bed, approached his bowl, approached him while the owner was cuddling him or while the cat was being pampered by the owner.

The cat approached amicably especially when the dog bent on the front limbs, lay down beside him or wagged his tail. The cat hissed or attacked when the dog approached with the tail up, entered his bed while he was sleeping, approached his bowl or approached him while the owner was cuddling him.

## Discussion

### Dog’s and cat’s relationship with people and other pets

The first part of the questionnaire investigated the owners’ opinions on the general relationship of the dog and cat living in the same household. Our results show that most owners perceived their dogs and cats as friendly with familiar people but having a different approach to strangers. Participants reported that in their dogs a sociable behaviour prevails although they are not always friendly with cats. Indeed, dogs were perceived as playful even with strangers and unknown conspecifics, while they could wag their tails or attack when seeing a cat. Conversely, owners perceived their cat as more disinterested in social relationships. Many participants referred that their cats have a fearful approach or are indifferent towards unknown people or conspecifics and, at the sight of dogs, run away or ignore them. A disagreement in the owner’s perception of their cohabiting pets as regards their response to unfamiliar people and animals also appeared, even though the pets shared the same social environment. Thus, the owner perceived the relationship with strangers or other pets of his/her cohabiting dog and cat as to be primarily a species-specific factor, regardless of their environment/family context. These findings are in agreement with Menchetti et al. [17] and Serpell [16], who described cats as more distant and neurotic (including aggressive, shy, and independent approaches) with strangers compared to dogs. Moreover, these findings support the public’s perception of the behaviour of the two species who believes dogs to be more extroverted and cats more independent and shy [15, 17, 23].

The dog's sociability and his interspecific communication skills are widely recognized, even by the observational studies. Several researches, comparing dogs’ social skills with those of wolves or chimpanzees, have emphasized the role of domestication and selective processes on dogs’ sociability [32, 33]. Dogs were the first animals to be domesticated and have shared a common ecological niche with humans for a longer time than any other species [1]. In reality, some authors hypothesized that humans and domestic dogs experienced convergent evolution of advanced social cognition, which has led to the emergence of one single, heterospecific group [34, 35]. Conversely, other authors [36–38] emphasized the role of learning in the development of dogs’ social skills for interacting with humans. As shown for a number of mammalian taxa [39], early social experiences affect later behaviour and the quality of relationship dogs and humans share, including aggressive and fearful attitudes [36, 40, 41]. In a 1961 study, Freedman et al. [38] showed that puppies that were not exposed to humans during their period of socialization were never able to develop normal bonds with humans later on. Scott and Fuller [42] obtained similar results. To conciliate the role of phylogeny and ontogeny, Udell et al. [43] supported the “Two Stage Hypothesis”. They proposed that the domestication *per se* has not necessarily changed the dog’s capacity to form social relationships, but it has extended the period of socialization, giving dogs more opportunities to form successful relations compared to other species [19]. In addition, it is well recognized that dogs can use their owner as a secure base [44] and form interspecific attachment bonds even in adulthood [45, 46].

For cats, the issue of sociability is even more controversial [17, 18, 47]. Contrary to the dog, the modern domestic cat is the product of a natural selection in which humans have intervened relatively recently (i.e. within the last 200 years; [1]). Feral and free-living domestic cats subsist by hunting alone and can live in the solitary state all their life [1, 18, 47]. The close coexistence of many cats is *in primis* conditioned by the food and space availability and appears to be the result of human intervention, for example for breeding purposes, in multiple-cat household or in colonies of abandoned cats [1, 18, 47, 48]. Unfamiliar cats are often turned away from a group and aggressive and abnormal behaviours are often reported within multiple-cat households [18, 49]. Potter and Mills [50], using an Ainsworth Strange Situation Test, argued that cats have not been domesticated long enough to show a preference towards human interaction as a focus of safety and security in the same way that dogs do. They concluded claiming that cats are typically quite autonomous in their social relationships [50].

However, cats can engage in a variety of intraspecific interactions and co-operative relationships so that the colony looks like a complex social organization [18, 47, 48]. The amount of affiliative behaviours results to be associated with familiarity and relatedness between cats [51, 52]. The cat appears to be also fit for an interspecific social system. Cats distinguish the voice of family and non-family members [22, 25] and can use human pointing to locate hidden food in a manner similar to the dog [23]. The cat has transferred some intraspecific behaviour, such as tail up, rubbing, kneading and purring, in the relationship towards humans [18]. Other communicative signals, like the “meow” vocalization, seem mainly used by pet cats to attract human attention [18, 24, 47, 48]. Contrary to Potter and Mills’ [50] claim, Edwards et al. [25] sustained that cats can manifest attachment behaviours towards their owners. Moreover, Vitale Shreve et al [21], using a free operant preference assessment, demonstrated that cats prefer social interaction with a human rather than other stimuli such as food, toys or scent. However, Vitale Shreve et al [21] admitted that there is a large individual variation on cats’ social behaviour, probably due to a combination of factors including biological predispositions and lifetime experiences. In fact, as for the dog, the cat's social behaviour is also affected by the amount of handling received during the socialisation period [48, 53].

Can we ultimately confirm the common beliefs about the sociability of dogs and cats? Can human's approach, perhaps not lacking in prejudices, influence the perception of their behaviours? These are unanswered questions. Humans’ assumptions that dogs are more friendly and emotional while cats are more independent may lead to a qualitatively different type of relationship with these species [15, 23]. Moreover, although our survey proposed questions related to precise and real situations, the answers could be influenced by the subjective perception of the owner [17, 24].

### Relationship between dogs and cats living in the same household

The second section of the questionnaire deepened the owner’s perception of the relationship between dogs and cats living in the same household. Our findings suggested that cats can develop interspecific social bonds provided there is a familiarity with each other. This bond emerged in social play and physical proximity of cats and dogs living in the same household although they both retain species-specific characteristics. Dogs and cats living together have different feeding and sleeping habits but show signs of mutual good relationship. Usually, dogs and cats eat in separate locations but, in a quarter of the dyads, the animal that first finishes his/her meal waits close to the animal who is still eating. Moreover, more than half of the respondents stated that their pets sleep and play together. Perhaps they are not best friends, but neither mortal enemies.

The most popular games that engage dogs and cats living together include making ambushes and chasing each other, the so-called “play-fighting”. This is not surprising since it is a typical intraspecific play in both cats and dogs [48, 49, 54]. In the domestic dog, play can be a result of the domestication, therefore humans selected them based on a general tendency towards playfulness. Moreover, it often involves a canine or human partner assuming a predominant social motivation: playing helps to establish, reinforce or test social bonds [26, 54, 55]. In particular, some authors [26, 54] sustain that dog-human play is a tool to form and maintain an emotionally-based bond.

Play for the cat is structurally and motivationally more similar to hunting behaviour since it involves often prey-like objects and exploratory behaviours [54]. However, there are playful interactions within colonies which continue in adult cats and a social motivation cannot be excluded even for the feline species [47, 48]. To our knowledge, there are no previous studies on play dynamics between dogs and cats. Moreover, numerous functions have been attributed to animal play [18, 55, 56]. “Play-fighting” may also be stressful [48, 55] and not always the distinction between play fighting and real fighting is clear [55]. Anyway, we considered the play between dogs and cats living together as a positive social indicator. Indeed, play behaviour has generally been identified as a potential welfare indicator since it is associated to positive emotions [57], it occurs when conditions are optimum and other needs are met [55, 57], it can turn a stranger into a familiar animal [56], and may indicate an interspecific attachment [26].

How do dogs and cats living together interact? Only 18% of owners stated that their pets ignore each other and not even 1% (9 owners) reported reciprocal aggressive interactions. Conversely, more than half of the owners referred that their dog and cat showed friendly interactions. However, there were differences in the dog and cat approaches, even though they lived in the same household. Owners reported that their dogs lick their roommate more frequently than cats while more cats ignore the cohabiting dog, move away or escape than vice versa. In the owner opinion, when the dog returns home from the walk, most cats ignore him while more often the dog approaches the cat wagging his/her tail or smelling the cat’s back. Then, a playful attitude of the cat towards his/her dog roommate prevails but the comparison between species shows a more frequent indifferent approach and minor interactivity for the cat. In the cat, the aggressive interactions towards the dog (hissing) are also greater than vice versa (growling), confirming the results of Thomson et al. [13].

As mentioned above, interspecific relationships in the cat are influenced by his evolutionary process, early socialization and later negative experiences, familiarity and individual personality [18, 48, 53]. In the Fox's experiment [58], some pups were raised with a litter of kittens together with their mother throughout the critical period of socialization. Fox [58] showed that cats not precociously socialized with dogs rarely interacted spontaneously with them, whereas the socialization with an alien species facilitated their social reactivity and playful interactions. In contrast, dog-raised dogs interact spontaneously with cats inviting them to play. In our survey, we can hypothesize that some cats exhibited few interactions with dogs due to poor familiarity or lack of early socialization. However, we found agreement on the age when dogs and cats living in the same household firstly met each other (in most pets, the first meeting occurred before six months). Unfortunately, we do not know the number of interspecific contacts during their socialisation period, as well as we do not have information about how long the pets of our survey have been cohabitating and which of the two species have been firstly introduced in the home. Anyway, species-specific personality traits seem to modulate the approach also between pets living together. Menchetti et al. [17], by using this same questionnaire, compared the owners’ perception of the personality traits of cats and dogs living in the same household finding dogs more sociable and protective whilst cats more neurotic. The results of this study, through a multifactorial approach, confirmed that learning and social context modulate the pet’s sociability but it is primarily a species-specific characteristic [17].

### Dogs and cats’ response to different approaches of the pets living with them

In the last part of the questionnaire, we analysed the reactions of dogs and cats, observed by the owners, in response to different postures and approaches of their roommate. Most respondents stated that their dogs and cats react amicably (“Wags tail”, “Get close amicably” or “Stays quiet”) in response to the roommate's different behaviours.

Tail wagging in dogs occurs in several situations. It can indicate excitement, stress [28, 59] or subordination [60], but usually communicates friendly intentions and an affectionate greeting [59–61]. Our results show that tail wagging mainly occurs when the cat bends on the front limbs, approaches him for a nose-to-nose greeting or with tail up and lies down beside him. Therefore, the dog sees these cat's attitudes as a positive signal to which he responds with sociability. Indeed, cats touch the nose and hold their tail up as a greeting and affiliative behaviours [18, 27, 48, 62]. Many dogs wag their tails even when the owner pampers the cat. In this context, tail wagging could also indicate stress and attention-seeking behaviour [28, 59]. Conversely, the owners reported that the dog reacts negatively, barks or attacks, mainly when the cat gets on his bed (even when it is empty), approaches his bowl, approaches while the owner is cuddling him. Then, some dogs also showed territorial and possessive attitudes. Moreover, differently from what Thomson et al. [13] report, we did not find a greater willingness of the dog to share his food than the cat.

Similarly, to the dog, the cat positively interpreted the dog bowing on the front limbs and lying by his side as well as the tail wagging. Again, the cat reacted negatively, hissing or attacking, when the dog entered his bed while he was sleeping (but not when the bed was empty) or approached his bowl. However, there were also many cats preferring to get away. The different findings obtained for the empty bed suggest that the cat, rather than possessive, could be annoyed by the presence of the dog and prefers to remain on his own.

Furthermore, unlike the dog, cats show aggressive behaviour when the dog approaches with the tail up. Then, we confirm that the tail position has different meanings in the species, but they understand each other. The tail held high is one of the dominant communicative features in the dog [60, 61, 63] and the cat can react negatively. Conversely, the vertical “tail up” is associated with intra and inter-specific affiliative behaviour in the cat signalling intention to interact amicably and inhibiting aggressiveness of the others [18, 27, 62]. Indeed, the dog’s response to the cat’s tail up was friendly.

Interestingly, the cat reacts negatively when the dog approaches while he is being pampered by the owner. This can suggest possessiveness and attachment to the owner similarly to the dog’s, confirming the results of Edwards et al. [25]. However, further studies about cat-owner relationship are needed.

### Limitations of the study

The present study investigated the behaviours of cats and dogs living together as perceived by the owner. The perception of the owner is an important aspect for understanding the human-animal relationships as it could influence the owner's approach to the two species. Moreover, the questionnaire is a widely used methodological approach to study behaviour and temperament of pets as owners can observe them in a variety of situations and over an extended period [17, 64]. However, the questionnaire inevitably involves a degree of subjectivity and some biases in the owners' responses. The voluntary participation in the questionnaire and the imbalance in the gender of participants could further aggravate these expected biases. For example, owners who are experiencing a peaceful coexistence of their animals may be more willing to fill in a questionnaire; in addition, women spend more time with their pets and generally tend to show more affective and empathic relationships [12]. Further sources of bias could be the popular breed stereotypes, owner’s personality, sensibility, and expectations as well as cultural aspects [12, 17, 64]. On the other hand, though, the large sample of owners and the possibility of examining the perception of a single owner on two paired animals supports the reliability of our results. Indeed, they agreed with most of the previous observational studies.

## Conclusions

Although there is no difference between dogs and cats in the relationship with familiar people, the approach to strangers and conspecifics is friendlier in dogs and more disinterested in cats. The dog has a more active approach with heterospecifics, with sociable or aggressive responses toward cats, while the cat prefers to ignore or escape at the sight of a dog. Species-specific features, different domestication processes and attitudes of people can motivate these differences. Dogs and cats retain their ethological characteristics of the species in living habits and social approaches, even when they share the same social and physical environment. The dog often looks for physical contact with the cat while the cat's approach is less interactive. However, cohabitation and familiarity allow the cat to form interspecific social bonds. Many dogs and cats living in the same household sleep and play together and few owners reported mutual aggressive interactions. Moreover, though the two species have different body languages, dogs and cats understand each other and respond accordingly: if the cat approaches with his tail up, the dog responds amicably while if the dog approaches with his tail up, the cat reacts with aggressive behaviours. A better understanding of the communicative signals of our pets would help improve management choices and relationship but observational studies should confirm our findings. Further studies should clarify the contribution of species-specific traits, learning abilities and owner's personality in the development of the pets’ sociability.

## Supporting information

S1 Table How pets interact with each other: Association between mutual aggressive interactions and demographic characteristics of owners and pets, and age at first encounter. (PDF)

S2 Table Relationship of the dog with the cat living in the same household. Values are number and percentage within Cat approach category in parentheses.
(PDF)

S3 Table Relationship of the cat with the dog living in the same household. Values are number and percentage within Dog approach and Cat reaction categories in parentheses.
(PDF)

S1 Database (XLSX)

S1 File Italian questionnaire. (PDF)

S2 File English questionnaire. (PDF)
