# Supplementary material for: Cats and dogs: Best friends or deadly enemies? What the owners of cats and dogs living in the same household think about their relationship with people and other pets
Source: PLoS One. 2020 Aug 26;15(8):e0237822. doi: 10.1371/journal.pone.0237822 (PMC7449504; doi:10.1371/journal.pone.0237822)
Supplement: S1 Table — (PDF) [file pone.0237822.s001.pdf]

S1 Table. How pets interact with each other: association between mutual aggressive interactions and demographic characteristics of owners and pets, and age at first encounter. Values are number and percentage of valid responses in parentheses.

**Parameter**		**No or only one pet attacks (n=1213)**	**The cat and the dog attack each other (n=6)**	χ^{2*}	**P***
**Age of respondent (class)**	**18–25 years**	211 (17.4%)	1 (16.7%)	0.340	1.000
	**26–40 years**	540 (44.6%)	3 (50.0%)		
	**41–55 years**	377 (31.1%)	2 (33.3%)		
	**56–70 years**	84 (6.9%)	0 (0.0%)		
**Sex of respondent**	**Male**	105 (8.7%)	2 (33.3%)	4.535	0.091
	**Female**	1107 (91.3%)	4 (66.7%)		
**Age of cat**	**0–6 months**	48 (4.0%)	1 (16.7%)	1.631	0.778
	**>6 months to 2 years**	306 (25.3%)	1 (16.7%)		
	**>2 to 8 years**	534 (44.1%)	2 (33.3%)		
	**>8 years**	322 (26.6%)	2 (33.3%)		
**Sex of cat**	**male**	591 (48.9%)	3 (50.0%)	0.295	0.445
	**Female**	618 (51.1%)	3 (50.0%)		
**Age of dog**	**0–6 months**	23 (1.9%)	0 (0.0%)	3.198	0.306
	**>6 months to 2 years**	241 (19.9%)	2 (33.3%)		
	**>2 to 8 years**	597 (49.3%)	3 (50.0%)		
	**>8 years**	349 (28.8%)	1 (16.7%)		
**Sex of dog**	**Male**	536 (44.4%)	2 (33.3%)	0.003	0.636
	**Female**	672 (55.6%)	4 (66.7%)		
**Age of dog at the first encounter with a cat**	**< 6 months**	664 (55.0%)	4 (66.7%)	1.301	0.636
	**6 months - 2 years**	246 (20.4%)	0 (0.0%)		
	**> 2 years**	297 (24.6%)	2 (33.3%)		
**Age of cat at the first encounter with a dog**	**< 6 months**	696 (58.3%)	3 (50.0%)	0.743	0.850
	**6 months - 2 years**	231 (19.3%)	1 (16.7%)		
	**> 2 years**	267 (22.4%)	2 (33.3%)		

* Chi-Square or Fisher's exact tests. The Exact significances were reported.
