# Supplementary material for: Cats and dogs: Best friends or deadly enemies? What the owners of cats and dogs living in the same household think about their relationship with people and other pets
Source: PLoS One. 2020 Aug 26;15(8):e0237822. doi: 10.1371/journal.pone.0237822 (PMC7449504; doi:10.1371/journal.pone.0237822)
Supplement: S2 Table — Values are number and percentage within Cat approach category in parentheses. (PDF) [file pone.0237822.s002.pdf]

S2 Table. Relationship of the dog with the cat living in the same household. Values are number and percentage within Cat approach category in parentheses.

Cat approach	Dog reaction						Total number of valid answers	Chi-Square Goodness of Fit Tests within Cat approach category	
	Moves away	Turns his head	Wags the tail	Stays quiet	Growls	Attacks		χ^2	P
**Bends on the front limbs**	99 (8.5%; 6.7%)	64 (5.5%; 7.8%)	***388 (33.5%; 10.5%)***	***580 (50.0%; 10.6%)***	16 (1.4%; 2.1%)	12 (1.0%; 3.1%)	1159	1435.9	<0.001
**Approaches for a nose-nose greeting**	102 (8.8%; 6.9%)	*76 (6.6%; 9.3%)*	***700 (60.5%; 18.9%)***	**245 (21.2%; 4.5%)**	19 (1.6%; 2.5%)	15 (1.3%; 3.8%)	1157	1782.3	<0.001
**Turns his head to one side**	*156 (14.0%; 10.6%)*	72 (6.5%; 8.8%)	167 (15.0%; 4.5%)	***699 (62.6%; 12.7%)***	10 (0.9%; 1.3%)	12 (1.1%; 3.1%)	1116	1820.8	<0.001
**Lies down beside**	95 (8.3%; 6.4%)	*97 (8.5%; 11.9%)*	***430 (37.5%; 11.6%)***	**491 (42.8%; 8.9%)**	15 (1.3%; 2.0%)	19 (1.7%; 4.9%)	1147	1180.8	<0.001
**Wags the tail**	*179 (15.6%; 12.1%)*	72 (6.3%; 8.8%)	**312 (27.3%; 8.4%)**	***549 (48.0%; 10.0%)***	17 (1.5%; 2.2%)	15 (1.3%; 3.8%)	1144	1145.3	<0.001
**Approaches with tail up**	*156 (13.7%; 10.6%)*	*77 (6.7%; 9.4%)*	***449 (39.3%; 12.2%)***	**408 (35.7%; 7.4%)**	31 (2.7%; 4.0%)	21 (1.8%; 5.4%)	1142	958.2	<0.001
**Comes in the dog's bed (empty)**	***231 (20.1%; 15.6%)***	32 (2.8%; 3.9%)	172 (14.9%; 4.7%)	***608 (52.8%11.1%)***	65 (5.6%; 8.5%)	*44 (3.8%; 11.3%)*	1152	1242.8	<0.001
**Comes in the dog's bed while he sleeps**	***197 (17.7%; 13.3%)***	*95 (8.5%; 11.6%)*	116 (10.4%; 3.1%)	***526 (47.2%; 9.6%)***	*134 (12.0%; 17.4%)*	*47 (4.2%; 12.0%)*	1115	812.2	<0.001
**Approaches the dog bowl**	*138 (11.7%; 9.3%)*	37 (3.1%; 4.5%)	99 (8.4%; 2.7%)	***515 (43.8%; 9.4%)***	***302 (25.7%; 39.3%)***	*86 (7.3%; 22.0%)*	1177	831.7	<0.001
**Approaches while the owner is cuddling the dog**	63 (5.4%; 4.3%)	*89 (7.6%; 10.9%)*	*336 (28.7%; 9.1%)*	***509 (43.4%; 9.3%)***	*108 (9.2%; 14.1%)*	*67 (5.7%; 17.1%)*	1172	875.9	<0.001
**He is pampered by the owner**	62 (5.3%; 4.2%)	*106 (9.1%; 13.0%)*	***526 (45.2%; 14.2%)***	**366 (31.4%; 6.7%)**	51 (4.4%; 6.6%)	*53 (4.6%; 13.6%)*	1164	1058.3	<0.001
**Chi-Square Goodness of Fit Tests within Dog reaction category** χ^2	225.1	73.7	1052.4	305.4	1091.7	182.3			
**P**	<0.001	<0.001	<0.001	<0.001	<0.001	<0.001			

In bold the prevailing behaviours within Cat approach category; in italics the prevailing behaviours within Dog reaction category (Chi-Square Goodness of Fit Tests)
