# Supplementary material for: Cats and dogs: Best friends or deadly enemies? What the owners of cats and dogs living in the same household think about their relationship with people and other pets
Source: PLoS One. 2020 Aug 26;15(8):e0237822. doi: 10.1371/journal.pone.0237822 (PMC7449504; doi:10.1371/journal.pone.0237822)
Supplement: S3 Table — Values are number and percentage within Dog approach and Cat reaction categories in parentheses. (PDF) [file pone.0237822.s003.pdf]

S3 Table. Relationship of the cat with the dog living in the same household. Values are number and percentage within Dog approach and Cat reaction categories in parentheses.

Dog approach		Cat reaction					Total number of valid answers	Chi-Square Goodness of Fit Tests within Dog approach category	
		Moves away	Gets close amicably	Stays quiet	Hisses	Attacks		χ^2	P
**Bends on the front limbs**		*199 (17.2%; 12.1%)*	***332 (28.7%; 13.6%)***	**563 (48.6%; 8.8%)**	52 (4.5%; 7.9%)	12 (1.0%; 8.4%)	1158	869.9	<0.001
**Turns his head to one side**		91 (8.1%; 5.5%)	153 (13.6%; 6.3%)	***846 (75.3%; 13.2%)***	24 (2.1%; 3.6%)	9 (0.8%; 6.3%)	1123	2207.6	<0.001
**Lies down beside**		83 (7.3%; 5.0%)	***431 (37.9%; 17.7%)***	**592 (52.0%; 9.2%)**	25 (2.2%; 3.8%)	7 (0.6%; 4.9%)	1138	1251.2	<0.001
**Wags the tail**		*94 (8.3%; 5.7%)*	***461 (40.5%; 18.9%)***	**542 (47.6%; 8.4%)**	38 (3.3%; 5.8%)	4 (0.4%; 2.8%)	1139	1128.7	<0.001
**Approaches with tail up**		*199 (17.8%; 12.1%)*	218 (19.4%; 9.0%)	**599 (53.4%; 9.3%)**	*89 (7.9%; 13.5%)*	*16 (1.4%; 11.2%)*	1121	904.4	<0.001
**Comes in the cat's bed (empty)**		*193 (17.4%; 11.7%)*	156 (14.0%; 6.4%)	***690 (62.1%; 10.7%)***	59 (5.3%; 9.0%)	14 (1.3%; 9.8%)	1112	1322.2	<0.001
**Comes in the cat's bed while he sleeps**		***292 (26.8%; 17.7%)***	116 (10.6%; 4.8%)	**502 (46.0%; 7.8%)**	*147 (13.5%; 22.3%)*	*34 (3.1%; 23.8%)*	1091	620.7	<0.001
**Approaches the cat bowl**		***237 (20.9%; 14.4%)***	70 (6.2%; 2.9%)	***705 (62.2%; 11.0%)***	*103 (9.1%; 15.6%)*	*19 (1.7%; 13.3%)*	1134	1375.1	<0.001
**Approaches while the owner is cuddling the cat**		*193 (16.7%; 11.7%)*	210 (18.2%; 8.6%)	***646 (55.9%; 10.0%)***	*91 (7.9%; 13.8%)*	*16 (1.4%; 11.2%)*	1156	1037.8	<0.001
**The dog is pampered by the owner**		70 (6.1%; 4.2%)	***288 (25.2%; 11.8%)***	***744 (65.0%; 11.6%)***	31 (2.7%; 4.7%)	12 (1.0%; 8.4%)	1145	1660.6	<0.001
**Chi-Square Goodness of Fit Tests within Cat reaction category**	χ^2	311.7	641.7	153.2	224.3	43.0			
	**P**	<0.001	<0.001	<0.001	<0.001	<0.001			

In bold the prevailing behaviours within Dog approach category; in italics the prevailing behaviours within Cat reaction category (Chi-Square Goodness of Fit Tests)
