# Supplementary material for: Cats and dogs: Best friends or deadly enemies? What the owners of cats and dogs living in the same household think about their relationship with people and other pets
Source: PLoS One. 2020 Aug 26;15(8):e0237822. doi: 10.1371/journal.pone.0237822 (PMC7449504; doi:10.1371/journal.pone.0237822)
Supplement: S1 File — (PDF) [file pone.0237822.s005.pdf]

DATI DEL PROPRIETARIO

SEZIONE A: DATI DEMOGRAFICI DEL PROPRIETARIO

Q1. Sesso
- o Maschio
- o Femmina

Q2. Età
- o 18–25 anni
- o 26–40 anni
- o 41–55 anni
- o 56–70 anni
- o

Q3. Regione
- o Nord (Valle d'Aosta, Piemonte, Liguria, Lombardia, Veneto, Trentino Alto Adige, Friuli Venezia Giulia, Emilia Romagna)
- o Centro (Toscana, Marche, Lazio, Umbria)
- o Sud (Campania, Abruzzo, Molise, Puglia, Basilicata, Calabria, Sicilia, Sardegna)

Q4. Sei un esperto? (veterinario, istruttore cinofilo, volontario protezione animale, allevatore, appassionato della materia, etc.)
- o Nessuna esperienza
- o Esperienza con cane
- o Esperienza con gatto
- o Esperienza con cane/gatto

Q5. N° animali con cui vivi _____

Q5.1. N° cani
- o 1 cane
- o 2–5 cani
- o >5 cani

Q5.2 N° gatti
- o 1 gatto
- o 2–5 gatti
- o >5 gatti

DATI DEL CANE

SEZIONE B: DATI DEMOGRAFICI

Q6. Età
- o 0–6 mesi
- o >6 mesi to 2 anni
- o >2 a 8 anni
- o >8 anni

Q7. Sesso

- Maschio
- Femmina

Q8. Stato riproduttivo

- Intero
- Castrato/sterilizzato

Q9. Quanto pesa il tuo cane?

- 1-3 kg
- 4-10 kg
- 10-20 kg
- 20-40 kg
- > 40 kg

Q10. Razza

- Meticcio
- Razza pura

Q11. Età di acquisizione

- 1–3 mesi
- 4 mesi a 1 anno
- 1–8 anni
- >8 anni

Q12. Dove vive il cane?

- Fuori casa
- Dentro casa
- Sia fuori che dentro casa

Q13. Dove dorme il cane?

- Libero fuori casa
- In uno spazio confinato
- Nell’area di casa
- Dentro casa
- In camera da letto
- Sul letto
- Altro

Q14. Incontri precedenti con i gatti: età al primo incontro

- < 6 mesi
- 6 mesi-2 anni
- 2 anni

Q15. Tempo trascorso con la madre (con la cucciolata?)

- < 1 settimana
- fino a 1 mese
- fino a 3 mesi
- > 3 mesi
- sconosciuto

SEZIONE C: COMPORTAMENTO DEL CANE

Q16. Come è il tuo cane quando interagisce con cani che conosce?

- Giocoso / Socievole
- Disinteressato
- Aggressivo
- Pauroso / Spaventato / Sospettoso
- Altro

Q17. Come è il tuo cane quando interagisce con cani che non conosce?

- Giocoso / Socievole
- Disinteressato
- Aggressivo
- Pauroso / Spaventato / Sospettoso
- Altro

Q18. Come è il tuo cane quando interagisce con persone conosciute?

- Giocoso / Socievole
- Disinteressato
- Aggressivo
- Pauroso / Spaventato / Sospettoso
- Altro

Q19. Come è il tuo cane quando interagisce con persone che non conosce?

- Giocoso / Socievole
- Disinteressato
- Aggressivo
- Pauroso / Spaventato / Sospettoso
- Altro

Q20. Il cane ha mai attaccato il gatto con cui vive o un altro gatto?

- Si
- No

Q21. Il cane è mai stato attaccato da un gatto?

- Si
- No

Q22. Quando il cane vede un gatto, il cane ...

- Lo ignora
- Scodinzola
- Abbaia
- Ringhia
- Lo insegue
- Lo attacca
- Scappa
- Altro

DATI DEL GATTO

SEZIONE D: DATI DEMOGRAFICI

Q23. Età
- 0–6 mesi
- >6 mesi a 2 anni
- >2 to 8 anni
- >8 anni

Q24. Sesso
- Maschio
- Femmina

Q25. Il gatto è sterilizzato/castrato?
- Si
- No

Q26. Razza
- Meticcio (incluso il gatto Europeo)
- Razza pura

Q27. Età di acquisizione
- 1–3 mesi
- 4 mesi to 1 anni
- 1–8 anni
- >8 anni

Q28. Dove vive il gatto?
- Fuori casa
- Dentro casa
- Sia fuori che dentro casa

Q29. Dove dorme il gatto?
- Libero fuori casa
- In uno spazio confinato
- Nell'area di casa
- Dentro casa
- In camera da letto
- Sul letto
- Altro

Q30. Incontri precedenti con i cani: età al primo incontro
- < 6 mesi
- 6 mesi-2 anni
- 2 anni

Q31. Tempo trascorso con la madre (con la cucciolata?)
- < 1 settimana
- fino a 1 mese

- fino a 3 mesi
- > 3 mesi
- Sconosciuto

SEZIONE E: COMPORTAMENTO DEL GATTO

Q32. Come definiresti il tuo gatto quando interagisce con gatti che conosce?
- Giocoso / Socievole
- Disinteressato
- Aggressivo
- Pauroso / Spaventato / Sospettoso
- Altro

Q33. Come definiresti il tuo gatto quando interagisce con gatti che non conosce?
- Giocoso / Socievole
- Disinteressato
- Aggressivo
- Pauroso / Spaventato / Sospettoso
- Altro

Q34. Come definiresti il tuo gatto quando interagisce con persone che conosce?
- Giocoso / Socievole
- Disinteressato
- Aggressivo
- Pauroso / Spaventato / Sospettoso
- Altro

Q35. Come definiresti il tuo gatto quando interagisce con persone che non conosce?
- Giocoso / Socievole
- Disinteressato
- Aggressivo
- Pauroso / Spaventato / Sospettoso
- Altro

Q36. Il gatto ha mai attaccato il cane con cui vive o un altro cane?
- Si
- No

Q37. Il gatto è mai stato attaccato da un cane?
- Si
- No

Q38. Quando il gatto vede un cane, il gatto ...
- Lo ignora
- Lo approccia in modo amichevole
- Soffia
- Lo attacca
- Lo osserva
- Scappa
- Altro

SEZIONE F: RAPPORTO TRA IL GATTO E IL CANE

Q39. Il cane e il gatto mangiano ...

- o Nella stessa ciotola
- o In ciotole vicine l'una all'altra
- o In ciotole posizionate distanti tra loro
- o Il cane mangia in una ciotola a terra mentre il gatto in una ciotola posta in alto
- o Altro

Q40. L'animale che termina per primo il suo pasto ...

- o Si allontana
- o Attende vicino all'animale che sta ancora mangiando
- o Spinge via l'altro animale e mangia il suo pasto
- o Cane e gatto mangiano insieme dalla ciotola in cui è rimasto il cibo
- o Cane e gatto mangiano separati
- o Altro

Q41. Cane e gatto giocano insieme?

- o Si
- o No

Q42. Se si, come? *(più di una risposta è possibile)*

- o Si rincorrono
- o Fanno la lotta
- o Il gatto gioca con la coda del cane
- o Si fanno delle imboscate

Q43. Il cane e il gatto dormono insieme…

- o Mai
- o Occasionalmente
- o Sempre

Relazione tra cane e gatto (cane verso gatto)

Q44. Come interagisce il cane con il gatto? *(più di una risposta è possibile)*

- o Lo lecca
- o Ci gioca
- o Lo ignora
- o Si muove con attenzione
- o Scappa
- o Ringhia
- o Lo attacca
- o Altro

Q45. Quando il cane rientra a casa dopo la passeggiata, come interagisce con il gatto? *(più di una risposta è possibile)*

- o Nessuna interazione
- o Si avvicina al posteriore per annusarlo

- Avvicina il proprio muso a quello del gatto (contatto naso-naso)
- Si avvicina scodinzolando
- Si sdraia a pancia all'aria
- Altro

Q46. Se il gatto:
(Seleziona il comportamento che assomiglia di più alla risposta comportamentale del tuo cane alla postura del gatto).

APPROCCIO DEL GATTO	REAZIONE DEL CANE					
	**Si allontana**	**Gira la testa**	**Scodinzola**	**Rimane tranquillo**	**Ringhia**	**Attacca**
**Si inchina sugli arti anteriori**	☐	☐	☐	☐	☐	☐
**Si avvicina per un saluto naso-naso**	☐	☐	☐	☐	☐	☐
**Gira la testa da un lato**	☐	☐	☐	☐	☐	☐
**Si sdraia di fianco**	☐	☐	☐	☐	☐	☐
**Scodinzola**	☐	☐	☐	☐	☐	☐
**Se il gatto si avvicina con la coda alzata, il cane:**	☐	☐	☐	☐	☐	☐
**Se il gatto entra nella cuccia vuota del cane, il cane:**	☐	☐	☐	☐	☐	☐
**Se il gatto entra nella cuccia del cane mentre questo vi riposa, il cane:**	☐	☐	☐	☐	☐	☐
**Se il gatto si avvicina alla ciotola del cane, il cane:**	☐	☐	☐	☐	☐	☐
**Se il gatto si avvicina mentre il proprietario sta coccolando il cane, il cane:**	☐	☐	☐	☐	☐	☐
**Se il gatto viene coccolato dal proprietario, il cane:**	☐	☐	☐	☐	☐	☐

<u>Relazione tra gatto e cane (gatto verso cane)</u>

Q47. Come interagisce il gatto con il cane? *(più di una risposta è possibile)*
- Lo lecca
- Ci gioca
- Lo ignora
- Si muove con attenzione
- Scappa
- Soffia
- Lo attacca
- Altro

Q48. Quando il cane rientra a casa dopo la passeggiata, come interagisce il gatto? *(più di una risposta è possibile)*

- Nessuna interazione
- Si avvicina al posteriore per annusarlo
- Avvicina il proprio muso a quello del cane (contatto naso-naso)
- Si avvicina con la coda alzata e diritta
- Si sdraia a pancia all'aria
- Altro

Q49. Se il cane:

(Seleziona il comportamento che assomiglia di più alla risposta comportamentale del tuo gatto alla postura del cane).

APPROCCIO DEL CANE	REAZIONE DEL GATTO				
	Si allontana	Si avvicina amichevole	Sta tranquillo	Soffia	Attacca
Si inchina sugli arti anteriori	☐	☐	☐	☐	☐
Si avvicina per un saluto naso-naso	☐	☐	☐	☐	☐
Gira la testa da un lato	☐	☐	☐	☐	☐
Si sdraia di fianco	☐	☐	☐	☐	☐
Scodinzola	☐	☐	☐	☐	☐
Se il cane entra nella cuccia vuota del gatto, il gatto:	☐	☐	☐	☐	☐
Se il cane entra nella cuccia dove il gatto riposa, il gatto:	☐	☐	☐	☐	☐
Se il cane si avvicina la ciotola del gatto, il gatto:	☐	☐	☐	☐	☐
Se il cane si avvicina mentre il proprietario sta coccolando il gatto, il gatto:	☐	☐	☐	☐	☐
Se il cane viene coccolato dal proprietario, il gatto	☐	☐	☐	☐	☐
