# Supplementary material for: Cats and dogs: Best friends or deadly enemies? What the owners of cats and dogs living in the same household think about their relationship with people and other pets
Source: PLoS One. 2020 Aug 26;15(8):e0237822. doi: 10.1371/journal.pone.0237822 (PMC7449504; doi:10.1371/journal.pone.0237822)
Supplement: S2 File — (PDF) [file pone.0237822.s006.pdf]

OWNER DATA

SECTION A: OWNER DEMOGRAPHIC DATA

Q1. Gender
- o Male
- o Female

Q2. Age
- o 18–25 years
- o 26–40 years
- o 41–55 years
- o 56–70 years

Q3. Region
- o North (Valle d'Aosta, Piemonte, Liguria, Lombardia, Veneto, Trentino Alto Adige, Friuli Venezia Giulia, Emilia Romagna)
- o Centre (Toscana, Marche, Lazio, Umbria)
- o South (Campania, Abruzzo, Molise, Puglia, Basilicata, Calabria, Sicilia, Sardegna)

Q4. Expert (veterinarian, trainer, animal-rights volunteer, breeder, enthusiast, etc...)
- o No expertise
- o Dogs expertise
- o Cats expertise
- o Dogs/Cats expertise

Q5. N° animals _____

Q5.1. N° dogs
- o 1 dog
- o 2–5 dogs
- o >5 dogs

Q5.2 N° cats
- o **1 cat**
- o **2–5 cats**
- o **>5 cats**

DOG DATA

SECTION B: DOG DEMOGRAPHIC DATA

Q6. Age
- o 0–6 months
- o >6 months to 2 years
- o >2 to 8 years
- o >8 years

Q7. Sex

- Male
- Female

Q8. Reproductive status

- Entire
- Neutered/spayed

Q9. How much weight the dog?

- 1-3 kg
- 4-10 kg
- 10-20 kg
- 20-40 kg
- > 40 kg

Q10. Breed

- Mixed
- Purebred

Q11. Age at acquisition

- 1–3 months
- 4 months to 1 year
- 1–8 years
- >8 years

Q12. Where does the pet live

- Outdoors
- Indoors
- Outdoors and indoors

Q13. Where does the pet sleep

- Free outdoors
- Enclosed space
- Home area
- Inside the house
- Bedroom
- On the bed
- Other

Q14. Previous meetings with cats: age at first encounter

- < 6 months
- 6months-2 years
- 2 years

Q15. Time spent with the mother (with the litter?)

- <1 week
- until 1 month
- until 3 months
- >3 months
- unknown

SECTION C: DOG BEHAVIOUR

Q16. How would you define your dog when interacting with known dogs?

- Playful / Sociable
- Uninterested
- Aggressive
- Fearful /Scary / Suspicious
- Other

Q17. How would you define your dog when interacting with unknown dogs?
- Playful / Sociable
- Uninterested
- Aggressive
- Fearful /Scary / Suspicious
- Other

Q18. How would you define your dog when interacting with known people?
- Playful / Sociable,
- Uninterested,
- Aggressive,
- Fearful /Scary / Suspicious,
- Other

Q19. How would you define your dog when interacting with unknown people?
- Playful / Sociable
- Uninterested
- Aggressive
- Fearful /Scary / Suspicious
- Other

Q20. Has the dog ever attacked the house cat or any another cat?
- Yes
- No

Q21. Has the dog ever been attacked by a cat?
- Yes
- No

Q22. When the dog sees a cat, the dog…
- Ignores it
- Wags his tail
- Barks
- Growls
- Chases it
- Attacks
- Runs away
- Other

CAT DATA

SECTION D: CAT DEMOGRAPHIC DATA

Q23. Age

- 0–6 months
- >6 months to 2 years
- >2 to 8 years
- >8 years

Q24. Sex

- Male
- Female

Q25. Is the cat neutered/spayed?

- Yes
- No

Q26. Breed

- Mixed (including European cats)
- Purebred

Q27. Age at acquisition

- 1–3 months
- 4 months to 1 year
- 1–8 years
- >8 years

Q28. Where the cat lives

- Outdoors
- Indoors
- Outdoors and indoors

Q29. Where the pet sleeps

- Free outdoors
- Enclosed space
- Home area
- Free inside the house
- Bedroom
- On the bed
- Other

Q30. When did the cat first meet with a dog?

- < 6 months
- 6 months-2years
- 2 years

Q31. Age spent with mother (with the litter?)

- <1 week

- Until 1 month
- Until 3 months
- >3 months
- Unknown

SECTION E: CAT BEHAVIOUR

Q32. How would you define your cat when interacting with known cats?
- Playful / Sociable
- Uninterested
- Aggressive
- Fearful /Scary / Suspicious
- Other

Q33. How would you define your cat when interacting with unknown cats?
- Playful / Sociable
- Uninterested
- Aggressive
- Fearful /Scary / Suspicious
- Other

Q34. How would you define your cat when interacting with known people?
- Playful / Sociable
- Uninterested
- Aggressive
- Fearful /Scary / Suspicious
- Other

Q35. How would you define your cat when interacting with known people?
- Playful / Sociable
- Uninterested
- Aggressive
- Fearful /Scary / Suspicious
- Other

Q36. Has the cat ever attacked the family dog or any other dog?
- Yes
- No

Q37. Has the cat ever been attacked by a dog?
- Yes
- No

Q38. When the cat sees a dog, the cat…:
- Ignores it
- Approaches him amicably
- Hisses
- Attacks
- Observes it
- Runs away
- Other

SECTION F: RELATIONSHIP BETWEEN THE CAT AND THE DOG

Q39. The dog and the cat eat…
- In the same bowl
- In bowls close to one another
- In bowls positioned far from each other
- The dog eats in a bowl placed on the ground while the cat in a bowl positioned at the top
- Other

Q40. The animal that first ends its meal…
- Moves away
- Waits close to the animal that is still eating
- Pushes away the other animal and eats his meal
- Dog and cat eat together from the bowl where food is remained
- Dog and cat eat separate
- Other

Q41. Do they play together?
- Yes
- No

Q42. If yes, how? (*more than one answer is possible*)
- chase each other
- fight
- cat plays with the dog's tail
- make ambushes

Q43. Dog and cat sleep nearby…
- Never
- Occasionally
- Always

Relationship between dog and cat (dog towards cat)

Q44. How does the dog interact with the cat? (*more than one answer is possible*)
- licks
- plays
- ignores
- moves carefully
- runs away
- growls
- attacks
- other

Q45. When the dog comes home after the walk, how does he interact with the cat? (*more than one answer is possible*)

- no interaction
- approaches the back to smell it
- nose contact
- approaches wagging
- lies down
- other

Q46. If the cat:

(Select the behaviour that most resembles your dog's response to the cat's posture).

	**DOG REACTION**					
**CAT APPROACH**	**Moves away**	**Turns his head**	**Wags the tail**	**Stays quiet**	**Growls**	**Attacks**
**Bends on the front limbs**	☐	☐	☐	☐	☐	☐
**Approaches for a nose-nose greeting**	☐	☐	☐	☐	☐	☐
**Turns his head to one side**	☐	☐	☐	☐	☐	☐
**Lies down beside**	☐	☐	☐	☐	☐	☐
**Wags the tail**	☐	☐	☐	☐	☐	☐
**Approaches with tail up**	☐	☐	☐	☐	☐	☐
**If the cat comes in the dog's bed (empty), the dog:**	☐	☐	☐	☐	☐	☐
**If the cat comes in the dog's bed while he sleeps, the dog:**	☐	☐	☐	☐	☐	☐
**If the cat approaches the dog bowl, the dog:**	☐	☐	☐	☐	☐	☐
**If the cat approaches while the owner is cuddling the dog, the dog:**	☐	☐	☐	☐	☐	☐
**If the cat is pampered by the owner, the dog:**	☐	☐	☐	☐	☐	☐

Relationship between dog and cat (cat towards dog)

Q47. How does the cat interact with the dog? (*more than one answer is possible*)

- licks
- plays
- ignores
- slowly moves
- runs away
- hisses
- attacks
- other

Q48. When the dog returns home from a walk, how the cat interacts with the dog? (*more than one answer is possible*)

- o no interaction
- o approaches the back to smell it
- o nose contact
- o approaches tail up
- o lies down
- o other

Q49. If the dog:

(Select the behaviour that most resembles your cat's response to the dog's posture).

	CAT REACTION				
**DOG APPROACH**	**Moves away**	**Gets close amicably**	**Stays quiet**	**Hisses**	**Attacks**
**Bends on the front limbs**	☐	☐	☐	☐	☐
**Turns his head to one side**	☐	☐	☐	☐	☐
**Lies down beside**	☐	☐	☐	☐	☐
**Wags the tail**	☐	☐	☐	☐	☐
**If the dog comes in the cat's bed (empty), the cat:**	☐	☐	☐	☐	☐
**If the dog comes in the cat's bed while he sleeps, the cat:**	☐	☐	☐	☐	☐
**If the dog approaches the cat bowl, the cat:**	☐	☐	☐	☐	☐
**If the dog approaches while the owner is cuddling the cat, the cat:**	☐	☐	☐	☐	☐
**If the dog is pampered by the owner, the cat:**	☐	☐	☐	☐	☐
